# Classification between Normal and Cancerous Human Urothelial Cells by Using Micro-Dimensional Electrochemical Impedance Spectroscopy Combined with Machine Learning

**DOI:** 10.3390/s22207969

**Published:** 2022-10-19

**Authors:** Ho-Jung Jeong, Kihyun Kim, Hyeon Woo Kim, Yangkyu Park

**Affiliations:** 1 Lighting Materials and Components Research Center, Korea Photonics Technology Institute (KOPTI), Gwangju 61007, Korea; 2 Department of Mechanical Design Engineering, Chonnam National University, 50 Daehak-ro, Yeosu 59626, Korea; 3 Department of Urology, Pusan National University Hospital, 179 Gudeok-ro, Seo-gu, Busan 49241, Korea; 4 Biomedical Research Institute, Pusan National University Hospital, 179 Gudeok-ro, Seo-gu, Busan 49241, Korea

**Keywords:** micro-dimensional electrochemical impedance spectroscopy (µEIS), machine learning, flow cytometry, urothelial cancer of the bladder (UBC)

## Abstract

Although the high incidence and recurrence rates of urothelial cancer of the bladder (UCB) are heavy burdens, a noninvasive tool for effectively detecting UCB as an alternative to voided urine cytology, which has low sensitivity, is yet to be reported. Herein, we propose an intelligent discrimination method between normal (SV-HUC-1) and cancerous (TCCSUP) urothelial cells by using a combination of micro-dimensional electrochemical impedance spectroscopy (µEIS) with machine learning (ML) for a noninvasive and high-accuracy UCB diagnostic tool. We developed a unique valved flow cytometry, equipped with a pneumatic valve to increase sensitivity without cell clogging. Since contact between a cell and electrodes is tight with a high volume fraction, the electric field can be effectively confined to the cell. This enables the proposed sensor to highly discriminate different cell types at frequencies of 10, 50, 100, 500 kHz, and 1 MHz. A total of 236 impedance spectra were applied to six ML models, and systematic comparisons of the ML models were carried out. The hyperparameters were estimated by conducting a grid search or Bayesian optimization. Among the ML models, random forest strongly discriminated between SV-HUC-1 and TCCSUP, with an accuracy of 91.7%, sensitivity of 92.9%, precision of 92.9%, specificity of 90%, and F1-score of 93.8%.

## 1. Introduction

Urothelial cancer of the bladder (UCB) is ranked as the tenth most commonly diagnosed cancer worldwide, with an estimated 573,000 new cases and 213,000 deaths each year [1]. The high incidence and recurrence rates of UCB are a heavy burden on both clinicians and patients. UCB is divided into non-muscle-invasive bladder cancer (NMIBC) and muscle-invasive bladder cancer (MIBC). NMIBC accounts for approximately 70% of all UCB cases and has a high recurrence rate (50–70% of patients) [2]. Local therapies can be administered to treat NMIBC; but, when NMIBC progresses to MIBC, the use of radical surgery and systemic therapy is inevitable [3]. Thus, early detection of initial or recurrent NMIBC before it progresses to MIBC is essential for reducing aggressive treatment. 

Cystoscopy is considered the gold standard for UCB detection. This technique can detect the presence of tumors by means of direct internal visualization of the bladder by using a cystoscope. Unfortunately, the invasiveness of cystoscopy can cause transient complications such as hematuria, urethral injury, and dysuria, as well as pain and discomfort during the procedure. Consequently, many patients are fearful and hesitant to undergo an examination for possible UCB [4]. This can hinder early detection of UCB, leading to mistimed diagnosis. Thus, effective noninvasive diagnostic methods are necessary for detecting initial-stage UBC and monitoring its recurrence.

An effective noninvasive diagnostic method to detect UCB has been an emerging field of interest. Voided urine cytology (VUC) is the most accepted noninvasive method [5]. However, while VUC has a high specificity of more than 90%, its sensitivity is lower than 40% [5]. Moreover, other noninvasive methods, such as the UroVysion fluorescence in situ hybridization (FISH) test and NMP22, have low accuracy [6,7]. Therefore, an alternative method that can detect cancers in voided urine with high accuracy is required to enhance the accessibility of bladder inspection. 

Electrochemical impedance spectroscopy (EIS), an analytical technique, can characterize the electrical properties of materials [8]. Impedance, which is the ratio of applied voltage to measured current in the frequency domain, is a complex number that can be described using the conjunction of resistance and reactance. Resistance and reactance are not constant, but depend on frequencies, and therefore, these signals provide a spectrum containing rich information about materials. Recently, machine learning (ML) has been applied to such impedance spectra to use them as fingerprints of materials for estimating target samples; for example, lithium-ion battery lifetime [9], metal corrosion [10], wood moisture content [11], milk adulteration [12], and health state of rolling bearing [13]. Furthermore, in the biomedical field, breast tissue [14,15] and prostate tissue [16] have been analyzed using combinations of impedance spectra and ML to detect subtle spectral patterns, and differentiate between normal and pathological tissues. 

To accurately measure impedance at the cellular level, a micro-dimensional electrochemical impedance spectroscopy (µEIS) chip, fabricated by means of a micromachining process, is necessary. µEIS can be divided into two types: single-cell trapping and flow cytometry. Single-cell trapping can provide an impedance spectrum with a wide frequency range. However, the limited throughput of this technique makes it inappropriate for ML. Moreover, single-cell trapping is quite laborious and time consuming because it is typically accompanied by examination under a microscope to confirm whether single cells are trapped. By contrast, flow cytometry easily measures the impedance of single cells in a flow state with a relatively high throughput, which may be suitable for clinical applications.

Several recent works have applied ML models to impedance cytometry data for aiding classification; for instance, k-means-based clustering of subpopulations of peripheral blood mononuclear cells [17], SVM to quantify eight groups of pollen grains [18], and several ML models for classification of apoptotic pancreatic cancer cell subpopulations [19]. Furthermore, neural networks have been utilized to characterize intrinsic properties, size, velocity, and cross-sectional position of cells [20,21].

In this study, we develop an accurate diagnostic method to discriminate between normal and cancerous human urothelial cells by using flow cytometry-based µEIS combined with ML. Additionally, we proposed valved flow cytometry, which is a µEIS equipped with a pneumatic valve to increase sensitivity without cell clogging. For measurement frequencies, 10, 100, and 500 kHz were selected to reflect characteristic frequencies of previous µEISs [22,23,24,25,26,27] with a concept similar to our sensor, such as tight electrical contact or constriction channels. In addition, 50 kHz and 1 MHz were added to increase feature dimension for ML. The impedance spectra were analyzed using various ML models, such as logistic regression (LR), k-nearest neighbor (KNN), decision tree (DT), random forest (RF), support vector machine (SVM), and backpropagation neural network (BPNN). The hyperparameters of each model were optimized by means of grid search or Bayesian optimization, and the prediction performance of the designed models are compared.

In contrast to a previous µEIS device with a pneumatic valve, which was used for single-cell trapping [24], our µEIS device is used for flow cytometry to achieve the high throughput required for ML analysis. Moreover, to the best of our knowledge, this is the first study in which ML is applied to µEIS spectra of urothelial cells, and no systematic comparisons of various ML models using µEIS spectra have yet been carried out. The fabrication of the proposed µEIS device, equipped with a pneumatic valve for flow cytometry, real-time impedance measurement, and ML-based impedance analysis are presented in this work. The present study is a step toward achieving our ultimate goal of noninvasively detecting UCB from urothelial cells within patients’ voided urine samples.

## 2. Materials and Methods

### 2.1. Materials

#### Cell Preparation

Normal (SV-HUC-1) and cancerous (TCCSUP) urothelial cell lines were cultured following the general guidelines of the American Type Culture Collection. We used F-12K medium for the SV-HUC-1 cells, and minimal essential medium (Eagle) in Earle’s balanced salt solution with non-essential amino acids and 1 mM sodium pyruvate for the TCCSUP cells. Both media were supplemented with fetal bovine serum, which accounted for 10% of the total volume. We incubated the cells in a 75 cm^2^ flask at 37 °C, subcultured them at intervals of 3–5 days, and changed the cell media 2–3 times per week. Phosphate-buffered saline (PBS) was used to suspend the cells, and cell density was maintained at approximately 4 × 10^6^/mL. The cell lines were manually stirred at 5-min intervals to ensure that they were uniformly distributed in the syringe. The room temperature was maintained at 22 °C throughout the experimental procedures.

### 2.2. Methods

#### 2.2.1. Device Configuration

**Figure 1:**
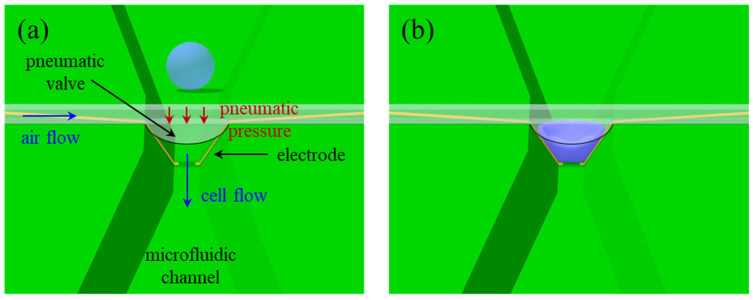
Configuration of the µEIS device equipped with a pneumatic valve for flow cytometry. (**a**) An unpressed cell before passing through the pneumatic valve, and (**b**) a cell squeezed by the pneumatic valve. Discrimination sensitivity can be enhanced on the basis of tight contact between the cell and electrode in the constricted channel.

The µEIS device for flow cytometry comprises a microfluidic channel with an inverted trapezoid shape, sensing electrodes on the slanted side walls of the channel, and a pneumatic valve to reduce the cross-sectional area of the channel, as illustrated in Figure 1. The valve is used to squeeze flowing cells, which allows elongated cells to be in tight contact with the sensing electrodes. Consequently, a high discrimination sensitivity can be expected. In contrast to constriction channel designs where the cross-sectional area of the channel is fixed [27,28], cell clogging issues in the µEIS with the valve can be solved easily by opening the pneumatic valve. Compared to other types of flow cytometry devices, where cells are floating in a medium [29], impedance variation due to differences in cell positions between the electrodes is less pronounced because the squeezed cells can maintain contact with the electrodes during impedance measurements. Moreover, the sensing electrodes are allowed to be formed easily in the microfluidic channel, which is explained in Section 2.2.2.

**Figure 2:**
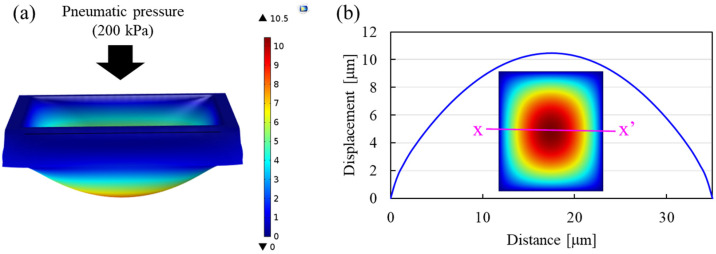
3D FEA for vertical-directional displacement of pneumatic valve. (**a**) Simulation results under the applied pressure of 200 kPa. (**b**) Displacement profile along cross-sectional line (xx’) in the inset that represents the nodal displacement of the bottom surface of the valve. The scale bar in (**a**) represents vertical-directional nodal displacement in both (**a**,**b**).

3D finite element analysis (FEA; COMSOL, Stockholm, Sweden) was performed to design the displacement of the valve, as shown in Figure 2a. A Mooney-Rivlin model was used to simulate hyperplastic behavior of polydimethylsiloxane (PDMS) under the applied pressure. Figure 2b shows the vertical-directional displacement along the cross-sectional line (xx’) in the inset that shows the nodal displacement of the bottom surface of the valve. The displacement at the center was about 10 µm under the applied pressure of 200 kPa, so that approximately 75% of the channel volume between the electrodes can be reduced.

**Figure 3:**
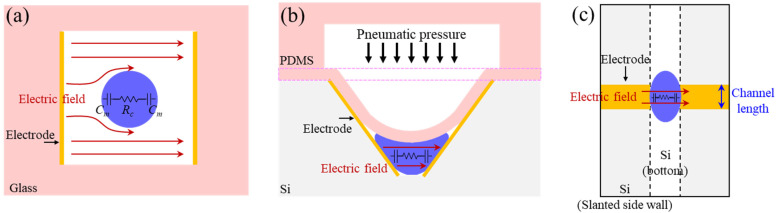
Comparison of electric field distribution between conventional and the proposed valved flow cytometry in low frequency range. Cross-sectional schematic images of (**a**) the conventional cytometry in which a cell floats in a medium, and (**b**) the valved cytometry with a squeezed cell. (**c**) Top view of the valved cytometry. A single cell is expressed by membrane capacitance (C_m_) in series with cytoplasm resistance (R_c_). The electric field of the conventional flow cytometry mainly passes through the medium, instead of the cell in the low frequency range. On the other hand, the electric field of the proposed cytometry can be confined into the membrane and cytoplasm of the cell due to the tight electrical contact between the cell and electrode, and the length of the electrode smaller than the squeezed cell. Thereby, the impedance signals measured by our sensor can effectively contain electrical properties of the cell.

Figure 3 compares electric field distribution of conventional and the proposed valved flow cytometry in low frequency range. In conventional flow cytometry, where a cell floats in a medium, as shown in Figure 3a, the electric field with a longer wavelength tends to pass through the medium, rather than the cell. On the other hand, the valve of our sensor was designed to squeeze the flowing cells (Figure 3b), and the electrode length (10 µm) was designed to be smaller than the squeezed cell length (Figure 3c). Accordingly, the squeezed cell will protrude out of the electrodes and cover almost all portions of the electrodes in tight contact, which means that the squeezed cell can fully occupy the volume between the electrodes. Thus, the electric field can penetrate the membrane and cytoplasm of the cell, even in the low frequency range, and the measured electrochemical impedance can effectively contain electrical properties of the cell. Indeed, several researchers reported that the electrical properties of cells were obtained within 1 MHz when a cell was in tight contact with electrodes [30,31,32,33]. Additionally, membrane capacitance and cytoplasm resistance were evaluated within 1 MHz using constriction channels for squeezing cells [25,27,34,35].

#### 2.2.2. Device Fabrication

**Figure 4:**
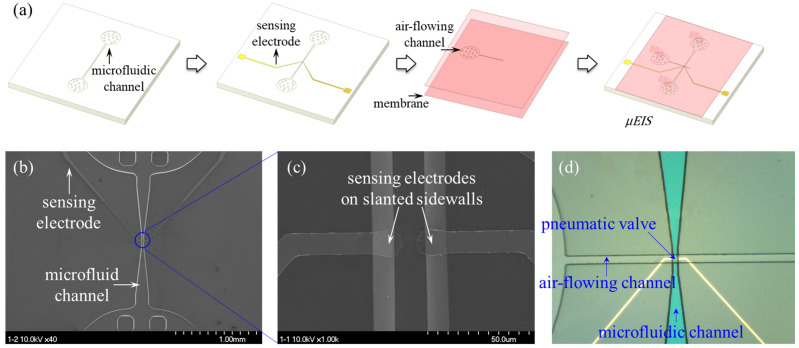
Fabrication of the µEIS device. (**a**) Schematics of fabrication flow. SEM images of (**b**) microfluidic channel integrated with sensing electrodes and (**c**) sensing electrodes on slanted sidewalls. (**d**) Optical microscopy image of the fabricated µEIS device.

The µEIS device for flow cytometry was fabricated from a silicon-based bulk and surface micromachining in combination with PDMS-based replica molding. Figure 4a shows the fabrication sequence of the µEIS device for flow cytometry.

Initially, a (100) silicon wafer was oxidized in a furnace. Next, photoresist (PR; GXR601) was spin-coated on the wafer and patterned by means of photolithography. Using the patterned PR, the thermal oxide layer was selectively etched by means of reactive ion etching. The oxide pattern was transferred onto the (100) silicon substrate, by means of anisotropic wet etching, to form a microfluidic channel with a trench depth of 15 µm. The channel was shaped as an inverted trapezoid along its cross-section view, and the slanted angle of the sidewalls was 54.74° because the lowest etching rate was in the direction of the <111> crystallographic surface.

After the wafer was oxidized again in a furnace, a negative PR (DNR-L300) was spin-coated onto it and patterned by means of photolithography. Then, Au/Cr was sputtered, and the PR was removed using acetone. Consequently, Au/Cr sensing electrodes were successfully formed, even on the slanted sidewalls. In the case of parallel electrodes, which are deposited on vertical sidewalls, each electrode was separately fabricated on a different substrate and aligned during the final fabrication step; alternatively complex fabrication, such as electroplating, was used [36,37]. By contrast, the slanted sidewalls of the µEIS device made it possible to easily form the sensing electrodes using general photolithography.

The pneumatic valve used consisted of an air-flowing channel and a flexible membrane. For the air-flowing channel, a patterned negative PR (SU-8 2025) was prepared as a mold. Subsequently, the PDMS replica was detached from the mold after baking in an oven. Then, PDMS was spin-coated on a wafer to obtain a flexible membrane. After plasma treatment of the PDMS membrane, the PDMS replica of the air-flowing channel was bonded to the membrane. By detaching the bonded PDMS from the wafer, the pneumatic valve was obtained. 

The top, bottom width, and height of the fluidic channel were 35, 15, and 15 µm, respectively, and the electrode width was 10 µm.

The pneumatic valve was bonded to the silicon substrate containing the microfluidic channel and sensing electrodes, which finally yielded the µEIS device for cytometry. Figure 4b–d shows scanning electron microscopy (SEM) and optical microscopy images of the µEIS device.

#### 2.2.3. Experiment Setup

The electrochemical impedances of the two groups of urothelial cells were measured by interfacing the µEIS device with a laptop, an impedance analyzer (HP 4294A, Agilent Technologies, Santa Clara, CA, USA), a syringe pump (Pump 11 Elite, Harvard Apparatus, Holliston, MA, USA), and a pneumatic pump (EFD 1500-XL, -Nordson EFD, Westlake, OH, USA). A voltage of 500 mV_pp_ was applied to the µEIS device. A flow rate of 0.1 µL/min from the syringe pump and a pneumatic pressure of 200 kPa from the pneumatic pump were applied in all the experiments. LabVIEW (National Instruments, Austin, TX, USA) was used to control the impedance analyzer and collect raw data in the form of text files in real time. 

#### 2.2.4. Data Analysis

The acquired impedance data were subjected to statistical analysis for comparing the impedances of the SV-HUC-1 and TCCSUP cells at each frequency. The differences were considered significant for *p*-values lower than 0.05. The statistical analysis was conducted in the PASW Statistics 18 software environment (IBM, Armonk, NY, USA).

The impedance spectra were transferred to a labeled dataset that was used to train and test various supervised ML models. Scikit-learn and the TensorFlow library of Python programming language were used in the analysis to implement the aforementioned models.

## 3. Results and Discussion

### 3.1. Electrochemical Impedance of Normal and Cancer Urothelial Cell Lines

**Figure 5:**
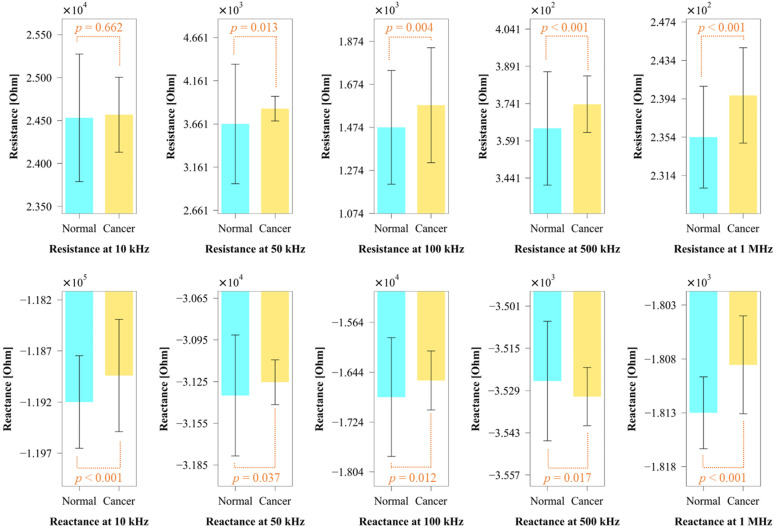
Impedance comparison between SV-HUC-1 and TCCSUP cells at each frequency. The blue and yellow bars represent the SV-HUC-1 and TCCSUP cells, respectively. The vertical error bars show standard deviation. All the impedance differences are statistically significant (*p* < 0.05), apart from the resistance at 10 kHz.

The cell suspensions of each of the cell lines were distributed into five 1-mL samples. The electrochemical impedances of the five samples of each of the cell lines were measured at five different frequencies (10, 50, 100, 500 kHz, and 1 MHz). In the normal state, the electrochemical impedance of the PBS was continually measured. When the target cells were squeezed by the pneumatic valve and passed through the sensing electrodes, distinct amplitude peaks were observed. Impedance responses with amplitudes greater than the offset value (m + 3σ) at each frequency were selected as the electrochemical impedance values of the cells. The m and σ in the offset value denote the mean and standard deviation, respectively, of the impedance amplitude of PBS. Approximately, more than 100 impedance values in each cell type were detected at each frequency. Student’s *t*-test was performed to compare the mean electrochemical impedance values of the two cell lines. Figure 5 depicts the average electrochemical impedance values of the two cell groups in terms of resistance and reactance. Our µEIS device well discriminated between the two cell lines. The differentiation was statistically significant at almost all frequencies, except in the case of resistance at 10 kHz. The statistical impedance difference, while useful, might be insufficient to distinguish between these cell types because of the overlapping impedance distribution. This could decrease the diagnostic accuracy and limit the use of the µEIS device in clinical settings.

The fully occupied cell between the electrodes allows the mainstream of the electric field to be confined to the membrane and cytoplasm of the cell, resulting in high sensitivity to reflect the electrical characteristics, such as membrane capacitance and cytoplasm resistance. Additionally, because the volume among the deflected valve and the two electrodes was designed to be much smaller than the average cell volume, the deviation of the volume fraction, depending on the cell size, could be reduced. This implies that electrical properties mainly contributed to the impedance difference between the respective cell types.

Using impedance detection methods of the tight contact or constriction channels, which are concepts similar to our sensor, several µEIS techniques detected other types of cancers or tumors. A µEIS device, in which the cells were trapped by negative pressure for the contact with electrodes, was introduced to discriminate between the normal and cancer human breast cells [22]. The electrochemical impedance was clearly discriminated at a frequency of 100 kHz. Another study reported a µEIS where the height of microfluidic channel was smaller than the cell diameter for the cell squeeze [23]. The optimal frequency, at which the impedance of two cell lines is most discriminated, was determined at 500 kHz. Prostate cancers were detected by a µEIS with a cell trap, using a constriction channel at optimal frequency of about 10 kHz [24]. Additionally, using another type of constriction channel, membrane capacitance and cytoplasm resistance of acute myeloid leukemia, human promyelocytic leukemia, kidney tumor, and lung cancer cells were quantified at 100 kHz [25,26,27]. In this study, 10, 100, and 500 kHz were selected to reflect their characteristic frequencies, and 50 kHz and 1 MHz were added to obtain more features for ML.

A few µEIS studies were carried out to detect urothelial cancer cells by means of cell trapping methods, and showed high discrimination capabilities [8,38]. Nevertheless, cell trapping methods can miss the cancer cells in the specimen due to the limitation of cell capturing, whereas our valved flow cytometry could promptly detect signals of almost all cells. Moreover, our method is more compatible than cell trapping method, especially in automated classification systems, since it is possible to measure more cells in a shorter time, without inspection for cell capturing. The experimental results of the referred studies [8,38] corresponded to our results: absolute amplitude and phase of impedance are lower for cancer cells.

Aside from urothelial cell level, a few studies measuring the electrical impedance of normal and cancerous urothelial tissues have been reported. The studies performed ex vivo and/or in vivo tests using EIS in bladder urothelial tissues to differentiate their pathologic diagnosis [39,40,41]; benign and malignant state was highly discriminated at the tissue level. However, those EIS require invasive procedures with a biopsy prior to the examination, whereas our study presents a possibility of only requiring the voided urine specimen. The noninvasiveness of our µEIS is one of the main advantages compared to the EIS devices applied in urothelial tissues.

### 3.2. Machine Learning

To prepare a dataset excluding a number (NaN) values, the number of feature vectors was determined as the least number of the cell impedance among the frequencies in each cell type. Specifically, 101 impedance values of the SV-HUC-1 cell line and 135 impedance values of the TCCSUP cell line at all frequencies were assembled, constituting 236 labeled impedance spectra composed of the impedance values recorded at five different frequencies. Consequently, the 236 feature vectors were used to feed the ML models. A total of 188 impedance spectra were randomly included in the training set to train and validate the models. The remaining 48 impedance spectra were included in the test set to evaluate the performance of these models. All the impedance spectra were subjected to z-score normalization preprocessing to maintain the same scale of the variables. Six ML models that are well known to be robust on classification tasks were selected; namely, LR, KNN, DT, RF, SVM, and BPNN. 

To alleviate the inherent overfitting risk of the ML models to the training set, K-fold cross-validation was performed with a K value of 10. After the training set was split into ten folds, nine folds were used for training, and the one remaining fold was used for model validation. The training and validation processes were repeated for ten folds (iterations), and the data in each fold were used once as the validation data. Then, the average validation error was calculated to identify the best parameters with the highest cross-validation accuracy. 

**Figure 6:**
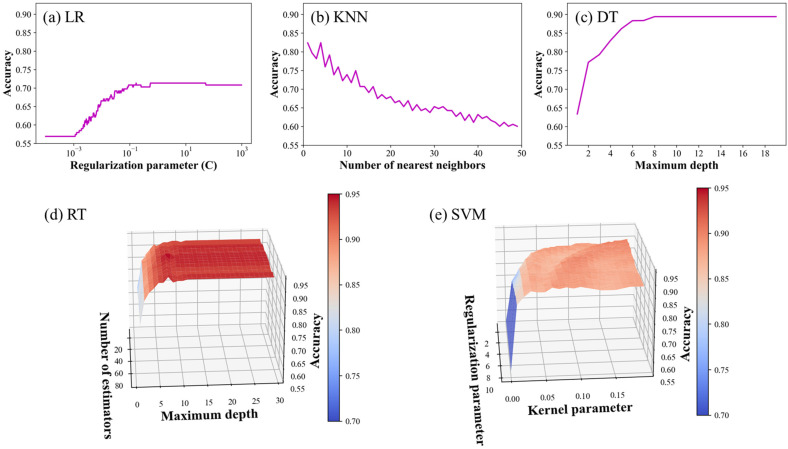
Hyperparameter optimization using grid search combined with ten-fold cross-validation for (**a**) LR, (**b**) KNN, (**c**) DT, (**d**) RT, and (**e**) SVM. The horizontal axis depicts the hyperparameters, and the vertical axis depicts the cross-validation accuracy. The color bars in (**d**,**e**), too, represent the cross-validation accuracy. The best hyperparameters are summarized in Table 1.

The hyperparameters of LR, KNN, DT, RF, and SVM were optimized by conducting a grid search in combination with the ten-fold cross-validation. A regularization parameter representing the inverse of the regularization strength was selected as the hyperparameter for LR. The number of nearest neighbors and the maximum depth were considered as the hyperparameters for KNN and DT, respectively. The maximum depth and the number of estimators were selected as the hyperparameters for RF, and the regularization parameter and kernel parameter were considered for SVM, where a radial basis function (RBF) was used as the kernel function. Figure 6 shows the cross-validation accuracy with respect to the corresponding hyperparameters during the iterative grid search. Among the five ML models, the cross-validation accuracy of the RT and SVM models exceeded 90%, with the best hyperparameters. The best hyperparameters were selected as follows: (maximum depth, number of estimators) for RT were (7, 50) and (regularization parameter, kernel parameter) for SVM were (2.12, 0.19). A more decisive hyperparameter can be determined from the gradient of the curved surface of the graph. The maximum depth was found to be more influential than the number of estimators in the case of RF, as shown in Figure 6d. In the case of SVM, as shown in Figure 6e, the kernel parameter was more critical than the regularization parameter in terms of the classification between the SV-HUC-1 and TCCSUP cell lines, by using the impedance spectra obtained from the µEIS device. 

**Table 1:** Optimization conditions and results of the six ML models.

	LR	KNN	DT	RF	SVM	BPNN *
Optimization method	Grid search	Grid search	Grid search	Grid search	Grid search	BO
Best Hyper -parameters	Regularization parameter: 0.17	Number of nearest neighbors: 4	Maximum depth: 5	Maximum depth: 7	Regularization parameter: 2.12	Batch size: 27
				Number of estimators: 50	Kernel parameter: 0.19	Learning rate: 0.0004
						Epoch: 329
Optimization time (seconds)	19.7	1.1	0.4	275.6	383.6	200.1
Best cross-validation accuracy	0.713	0.824	0.898	0.951	0.909	0.905

**Figure 7:**
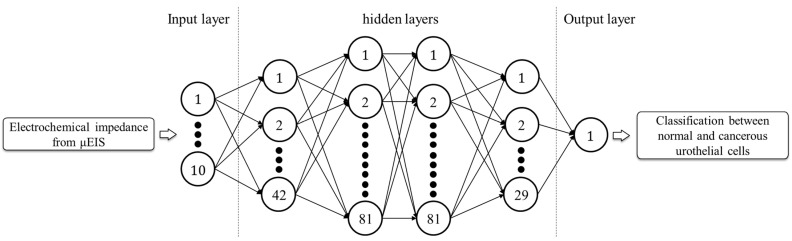
Hyperparameter optimization using BO with ten-fold cross-validation for BPNN. The number of layers and neurons that led to the best cross-validation accuracy of BPNN are shown. The number in the last neuron of each layer represents the number of neurons comprising the layer.

* The best hyperparameters for the number layers and neurons are depicted in Figure 7.

Different from the ML abovementioned models, the hyperparameters for network architecture, such as depth of layers and number of neurons, should be included for BPNN before compiling it into a model. For this reason, BPNN has a substantial number of hyperparameters. Therefore, BPNN would inevitably incur a considerable computational cost, as it attempts to determine the hyperparameters by conducting a grid search. Bayesian optimization (BO) can be used to tune hyperparameters in relatively few iterations by building a surrogate model based on the Gaussian process [42]. It is especially suitable for models whose hyperparameters are large in number, so that the computation to optimize them is expected to be expensive [43]. To effectively tune the hyperparameters, BO with ten-fold cross-validation was employed for BPNN in this study. The Adam optimizer, ReLU activation function in the hidden layers, and sigmoid function in the output layer were used. The number of hidden layers, learning rate, batch size, epoch, and number of neurons in each hidden layer were optimized as the BPNN hyperparameters. Figure 7 shows the determined BPNN architecture for a given depth of layers and number of neurons in each layer. The optimization time for the best parameters when using BO (200.1 s) was considerably shorter than those when using RF (275.6 s) and SVM (383.6 s), although the number of hyperparameters of BPNN was considerably greater. The best cross-validation accuracy for BPNN was 0.905, similar to that of SVM. Table 1 summarizes the optimization conditions and the results obtained for the six ML models. RF yielded the highest cross-validation accuracy among the ML models. 

**Figure 8:**
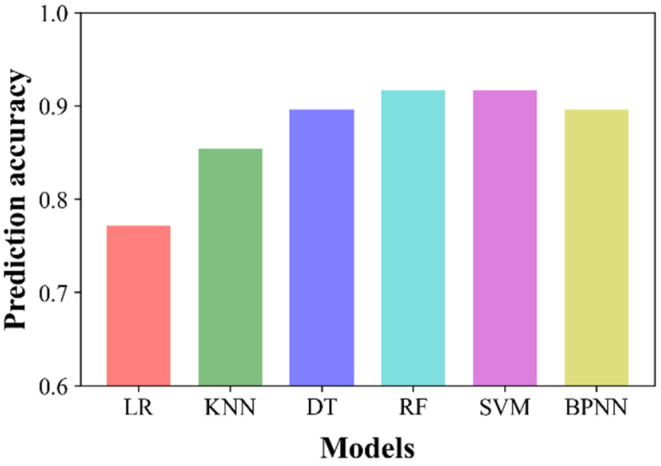
Prediction accuracy results of the six ML models. Both RF and SVM yielded the highest accuracy of 0.917.

Prediction accuracy on the test data was evaluated, as depicted in Figure 8. As expected in the cross-validation analysis, RF yielded the highest prediction accuracy of 0.917. Additionally, the accuracy of SVM was identical to that of RF, followed by BPNN and DT with an accuracy of 0.896, KNN, and LR. Prediction accuracies higher than 90% were obtained for RF and SVM among the six ML models. The performance of BPNN was slightly inferior to that of RF and SVM, probably because of the insufficient number of impedance spectra available for obtaining predictive information [44]. The linear LR classifier yielded the lowest accuracy, which indicates that the nonlinear classifiers were more appropriate for the impedance spectra obtained using the µEIS device compared to linear classifiers. Meanwhile, tree-based learning models such as DT and RF yielded good accuracy. For example, although DT was calibrated using only one hyperparameter, its prediction accuracy was comparable to that of BPNN. This implies that precise optimization using greater numbers of hyperparameters, such as the minimum number of samples to split, decision criteria, and the minimum number of samples at leaves, could improve the accuracy of DT and RF. 

**Figure 9:**
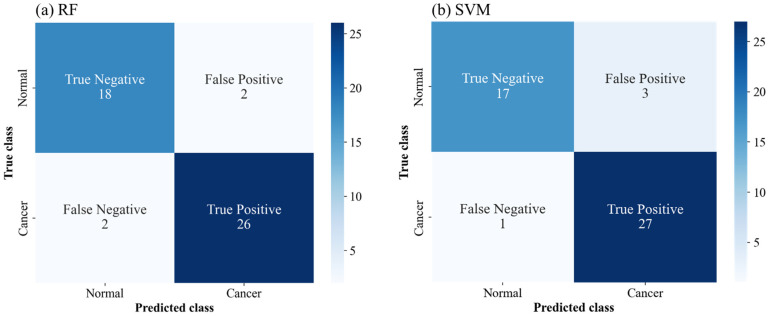
Confusion matrices on the test data for (**a**) RF and (**b**) SVM. RF is more precise and specific compared to SVM, while SVM has the superior F1 score.

Confusion matrices of the test data were obtained to evaluate performance parameters, such as sensitivity, precision, specificity, and F1 score, in addition to the accuracy of the ML models [44]. Figure 9 depicts a graphical representation of the confusion matrices of RF and SVM, which yielded the best prediction accuracy. The overall accuracy of RF and SVM were the same, but the elements in the corresponding matrices were different. This facilitated a stricter performance comparison. RF was found to be superior to SVM in terms of precision and specificity, while SVM was superior in terms of the F1 score. Table 2 summarizes the performance parameter scores for all the models. The best scores for each of the performance parameters are denoted by asterisks. According to the table, the most appropriate models are different for each performance parameter. For instance, KNN was the best model in terms of sensitivity (100%), in spite of its relatively low accuracy. Meanwhile, RF exhibited the highest performance in three of the five performance parameters. Notably, the sensitivity of RF was 0.929, considerably higher than that of the conventional VUC, while the specificity of RF was 0.9, comparable to that of the VUC. Furthermore, the application of various ML models to enhance specific performance scores can improve diagnostic performance. These results imply that the proposed method of combining µEIS and ML can provide a new noninvasive tool for detecting UCB by using voided urine specimens. 

In this study, we proved the effectiveness of ML in differentiating between the SV-HUC-1 and TCCSUP cell lines on the basis of their electrochemical impedance data measured using the µEIS device. Before applying the ML-µEIS combination method in actual clinical practice, studies evaluating the functional effectiveness of the µEIS device should be conducted by using actual urine specimens from UCB patients. A few supplementary techniques are required for successful evaluation of the device by using a urine specimen: (1) a filtration system to filter out other components within a urine specimen, such as red and white blood cells, from the urothelial cells; and (2) a cell-collection system that can retrieve urothelial cells from 24-h urine collection. 

Case-controlled studies using a large number of urine specimens should be conducted to validate the effectiveness of the µEIS-ML combination in detecting UCB. Moreover, determining the correlation between the impedance of urothelial cancer cells and the pathological grade of UCB would be useful for setting up treatment plans and predicting the prognosis of UCB patients. Additionally, a combination method of clustering by unsupervised learning and classification by supervised learning with each population being clustered [19] will be helpful in determining the discrimination of the minority subpopulation in a heterogeneous sample.

A limitation of this study is that low-grade urothelial cancer cells were not included in the analysis. Therefore, it is vague as to whether the electrochemical impedance values of normal and low-grade urothelial cancer cells significantly differ. Because low-grade urothelial cells present fewer deformities than high-grade cells, impedance may not show significant difference between normal and low-grade urothelial cancer cells. However, by applying ML with an abundant dataset, good levels of discrimination between normal and low-grade urothelial cancer cells could be expected by using their electrochemical impedance data. Additional studies should be conducted by including low-grade urothelial cancer cells to validate the effectiveness of ML. Once the abovementioned refinements and studies are conducted, the µEIS–ML combination can emerge as an effective noninvasive method for UCB detection.

The versatility of our sensor, such as high impedance sensitivity and being free from the cell clogging, can be widely applied to detect various cancer cell, such as leukemia, breast, lung, and prostate cancer cells, by measuring the impedance changes originated from the mutation of membrane capacitance or cytoplasm resistance. Additionally, it could be used to electrically characterize microbes and stem cells through a slight modification of the current sensor design and measurement setup. The channel size tunability of our sensor will further expand its application area.

## 4. Conclusions

In this study, we introduced the combination of µEIS with ML to discriminate between normal and cancerous human urothelial cells as a highly accurate and noninvasive diagnostic tool for detecting bladder cancer. Additionally, the valved flow cytometry was proposed to increase sensitivity and prevent cell clogging. Details pertaining to the fabrication of the proposed µEIS device, equipped with a pneumatic valve, real time impedance measurement, and impedance analysis using various ML models, were presented. The impedance differences were statistically significant at almost all the measurement frequencies (*p* < 0.05). The impedance spectra were applied to the various ML models, and systematic comparisons of the ML models were performed through validation and test processes. Hyperparameter optimization was conducted by means of a grid search and BO; the optimization time was much less for BPNN (200.1 s) using BO than RF (275.6 s) and SVM (383.6 s) using grid search. Among the ML models, RF performed the best on three of the five performance parameters, and it classified normal and cancer cells with an accuracy of 91.7%, sensitivity of 92.9%, precision of 92.9%, specificity of 90.0%, and F1-score of 93.8%. Accordingly, the µEIS-ML combination could emerge as a noninvasive diagnostic technique for UCB.

## Figures and Tables

**Table 2:** Comparison of performance parameters of the six ML models.

	LR	KNN	DT	RF	SVM	BPNN
Accuracy	0.771	0.854	0.896	0.917 *	0.917 *	0.896
Sensitivity	0.851	1.000 *	0.929	0.929	0.964	0.958
Precision	0.774	0.800	0.897	0.929 *	0.900	0.852
Specificity	0.650	0.650	0.850	0.900 *	0.850	0.833
F1-score	0.814	0.889	0.912	0.929	0.931 *	0.902

* Indicates the best score in each row.

## Data Availability

Not applicable.

## References

[1] Sung H., Ferlay J., Siegel R.L., Laversanne M., Soerjomataram I., Jemal A., Bray F. (2021). Global cancer statistics 2020: GLOBOCAN estimates of incidence and mortality worldwide for 36 cancers in 185 countries. CA A Cancer J. Clin..

[2] Lotan Y., Bivalacqua T.J., Downs T., Huang W., Jones J., Kamat A.M., Konety B., Malmström P.-U., McKiernan J., O’Donnell M. (2019). Blue light flexible cystoscopy with hexaminolevulinate in non-muscle-invasive bladder cancer: Review of the clinical evidence and consensus statement on optimal use in the USA—Update 2018. Nat. Rev. Urol..

[3] Van Rhijn B.W., Burger M., Lotan Y., Solsona E., Stief C.G., Sylvester R.J., Witjes J.A., Zlotta A.R. (2009). Recurrence and progression of disease in non–muscle-invasive bladder cancer: From epidemiology to treatment strategy. Eur. Urol..

[4] Van Der Aa M.N., Steyerberg E.W., Sen E.F., Zwarthoff E.C., Kirkels W.J., Van Der Kwast T.H., Essink-Bot M.L. (2008). Patients’ perceived burden of cystoscopic and urinary surveillance of bladder cancer: A randomized comparison. BJU Int..

[5] Carmack A.J., Soloway M.S. (2006). The diagnosis and staging of bladder cancer: From RBCs to TURs. Urology.

[6] Hajdinjak T. (2008). UroVysion FISH test for detecting urothelial cancers: Meta-analysis of diagnostic accuracy and comparison with urinary cytology testing. Urol. Oncol..

[7] Vrooman O.P., Witjes J.A. (2008). Urinary markers in bladder cancer. Eur. Urol..

[8] Park Y., Kim H.W., Yun J., Seo S., Park C.-J., Lee J.Z., Lee J.-H. (2016). Microelectrical impedance spectroscopy for the differentiation between normal and cancerous human urothelial cell lines: Real-time electrical impedance measurement at an optimal frequency. BioMed Res. Int..

[9] Zhang Y., Tang Q., Zhang Y., Wang J., Stimming U., Lee A.A. (2020). Identifying degradation patterns of lithium ion batteries from impedance spectroscopy using machine learning. Nat. Commun..

[10] Varvara S., Berghian-Grosan C., Bostan R., Ciceo R.L., Salarvand Z., Talebian M., Raeissi K., Izquierdo J., Souto R.M. (2021). Experimental characterization, machine learning analysis and computational modelling of the high effective inhibition of copper corrosion by 5-(4-pyridyl)-1,3,4-oxadiazole-2-thiol in saline environment. Electrochim. Acta.

[11] Daassi-Gnaba H., Oussar Y., Merlan M., Ditchi T., Géron E., Holé S. (2017). Wood moisture content prediction using feature selection techniques and a kernel method. Neurocomputing.

[12] Durante G., Becari W., Lima F.A., Peres H.E. (2015). Electrical impedance sensor for real-time detection of bovine milk adulteration. IEEE Sens. J..

[13] Kirchner E., Bienefeld C., Schirra T., Moltschanov A. (2022). Predicting the Electrical Impedance of Rolling Bearings Using Machine Learning Methods. Machines.

[14] Daliri M.R. (2015). Combining extreme learning machines using support vector machines for breast tissue classification. Comput. Methods Biomech. Biomed. Eng..

[15] Helwan A., Idoko J.B., Abiyev R.H. (2017). Machine learning techniques for classification of breast tissue. Procedia Comput. Sci..

[16] Murphy E.K., Mahara A., Khan S., Hyams E.S., Schned A.R., Pettus J., Halter R.J. (2017). Comparative study of separation between ex vivo prostatic malignant and benign tissue using electrical impedance spectroscopy and electrical impedance tomography. Physiol. Meas..

[17] Schütt J., Sandoval Bojorquez D.I., Avitabile E., Oliveros Mata E.S., Milyukov G., Colditz J., Delogu L.G., Rauner M., Feldmann A., Koristka S. (2020). Nanocytometer for smart analysis of peripheral blood and acute myeloid leukemia: A pilot study. Nano Lett..

[18] D’Orazio M., Reale R., De Ninno A., Brighetti M.A., Mencattini A., Businaro L., Martinelli E., Bisegna P., Travaglini A., Caselli F. (2021). Electro-Optical Classification of Pollen Grains via Microfluidics and Machine Learning. IEEE Trans. Biomed. Eng..

[19] Honrado C., Salahi A., Adair S.J., Moore J.H., Bauer T.W., Swami N.S. (2022). Automated biophysical classification of apoptotic pancreatic cancer cell subpopulations by using machine learning approaches with impedance cytometry. Lab Chip.

[20] Feng Y., Cheng Z., Chai H., He W., Huang L., Wang W. (2022). Neural network-enhanced real-time impedance flow cytometry for single-cell intrinsic characterization. Lab Chip.

[21] Honrado C., McGrath J.S., Reale R., Bisegna P., Swami N.S., Caselli F. (2020). A neural network approach for real-time particle/cell characterization in microfluidic impedance cytometry. Anal. Bioanal. Chem..

[22] Han A., Yang L., Frazier A.B. (2007). Quantification of the heterogeneity in breast cancer cell lines using whole-cell impedance spectroscopy. Clin. Cancer Res..

[23] Kang G., Yoo S.K., Kim H.-I., Lee J.-H. (2011). Differentiation between normal and cancerous cells at the single cell level using 3-D electrode electrical impedance spectroscopy. IEEE Sens. J..

[24] Kang G., Kim Y.-j., Moon H.-s., Lee J.-W., Yoo T.-K., Park K., Lee J.-H. (2013). Discrimination between the human prostate normal cell and cancer cell by using a novel electrical impedance spectroscopy controlling the cross-sectional area of a microfluidic channel. Biomicrofluidics.

[25] Zhao Y., Chen D., Li H., Luo Y., Deng B., Huang S.-B., Chiu T.-K., Wu M.-H., Long R., Hu H. (2013). A microfluidic system enabling continuous characterization of specific membrane capacitance and cytoplasm conductivity of single cells in suspension. Biosens. Bioelectron..

[26] Zhao Y., Chen D., Luo Y., Li H., Deng B., Huang S.-B., Chiu T.-K., Wu M.-H., Long R., Hu H. (2013). A microfluidic system for cell type classification based on cellular size-independent electrical properties. Lab Chip.

[27] Zheng Y., Shojaei-Baghini E., Wang C., Sun Y. (2013). Microfluidic characterization of specific membrane capacitance and cytoplasm conductivity of singlecells. Biosens. Bioelectron..

[28] Zhao Y., Jiang M., Chen D., Zhao X., Xue C., Hao R., Yue W., Wang J., Chen J. (2016). Single-cell electrical phenotyping enabling the classification of mouse tumor samples. Sci. Rep..

[29] Petchakup C., Li K.H.H., Hou H.W. (2017). Advances in single cell impedance cytometry for biomedical applications. Micromachines.

[30] Hong J.-L., Lan K.-C., Jang L.-S. (2012). Electrical characteristics analysis of various cancer cells using a microfluidic device based on single-cell impedance measurement. Sens. Actuators B Chem..

[31] Guo X., Zhu R., Zong X. (2015). A microchip integrating cell array positioning with in situ single-cell impedance measurement. Analyst.

[32] Jang L.-S., Wang M.-H. (2007). Microfluidic device for cell capture and impedance measurement. Biomed. Microdevices.

[33] Tsai S.L., Wang M.H., Chen M.K., Jang L.S. (2014). Analytical and numerical modeling methods for electrochemical impedance analysis of single cells on coplanar electrodes. Electroanalysis.

[34] Huang S.-B., Zhao Y., Chen D., Lee H.-C., Luo Y., Chiu T.-K., Wang J., Chen J., Wu M.-H. (2014). A clogging-free microfluidic platform with an incorporated pneumatically driven membrane-based active valve enabling specific membrane capacitance and cytoplasm conductivity characterization of single cells. Sens. Actuators B Chem..

[35] Chen J., Zheng Y., Tan Q., Zhang Y.L., Li J., Geddie W.R., Jewett M.A., Sun Y. (2011). A microfluidic device for simultaneous electrical and mechanical measurements on single cells. Biomicrofluidics.

[36] Frankowski M., Simon P., Bock N., El-Hasni A., Schnakenberg U., Neukammer J. (2015). Simultaneous optical and impedance analysis of single cells: A comparison of two microfluidic sensors with sheath flow focusing. Eng. Life Sci..

[37] Spencer D., Caselli F., Bisegna P., Morgan H. (2016). High accuracy particle analysis using sheathless microfluidic impedance cytometry. Lab Chip.

[38] Chuang C.-H., Huang Y.-W., Wu Y.-T. (2011). System-level biochip for impedance sensing and programmable manipulation of bladder cancer cells. Sensors.

[39] Keshtkar A., Keshtkar A., Smallwood R.H. (2006). Electrical impedance spectroscopy and the diagnosis of bladder pathology. Physiol. Meas..

[40] Wilkinson B., Smallwood R., Keshtar A., Lee J., Hamdy F. (2002). Electrical impedance spectroscopy and the diagnosis of bladder pathology: A pilot study. J. Urol..

[41] Keshtkar A., Salehnia Z., Keshtkar A., Shokouhi B. (2012). Bladder cancer detection using electrical impedance technique (Tabriz Mark 1). Pathol. Res. Int..

[42] Močkus J. (1975). On Bayesian methods for seeking the extremum. Optimization Techniques, Proceedings of the IFIP Technical Conference, Novosibirsk, Russia, 1–7 July 1974.

[43] Itakura K., Saito Y., Suzuki T., Kondo N., Hosoi F. (2018). Estimation of citrus maturity with fluorescence spectroscopy using deep learning. Horticulturae.

[44] Schackart III K.E., Yoon J.-Y. (2021). Machine learning enhances the performance of bioreceptor-free biosensors. Sensors.

